# Evaluation of the antiviral activity of ultraviolet light and zinc oxide nanoparticles on textile products exposed to *Avian coronavirus*

**DOI:** 10.1038/s41598-023-36100-9

**Published:** 2023-06-14

**Authors:** David Asmat-Campos, Jesús Rojas-Jaimes, Eliana Icochea-D’Arrigo, Gina R. Castro-Sanguinetti, Juan Anderson More-Bayona, Luisa Juárez-Cortijo, Daniel Delfín-Narciso, Gabriela Montes de Oca-Vásquez

**Affiliations:** 1 Dirección de Investigación, Innovación y Responsabilidad Social, Universidad Privada del Norte, Trujillo, Peru; 2 Laboratorio de Patología Aviar, Facultad de Medicina Veterinaria, Universidad Nacional Mayor de San Marcos, Lima, Peru; 3 Grupo de Investigación en Ciencias Aplicadas y Nuevas Tecnologías, Universidad Privada del Norte, 13011 Trujillo, Peru; 4 National Laboratory of Nanotechnology, National Center for High Technology, Pavas, San José, 10109 Costa Rica

**Keywords:** Microbiology, Nanoscience and technology

## Abstract

This research has developed a piece of sanitizing locker-model equipment for textiles exposed to avian coronavirus, which has been put under the influence of UV light, UV + zinc oxide nanoparticles (phytosynthesized ZnONP), and water + UV, and, in turn, under the influence of the exposure time (60, 120, 180 s). The results linked to the phytosynthesis of ZnONP indicate a novel method of fabricating nanostructured material, nanoparticles with spherical morphology and an average size of 30 nm. The assays were made based on the viral viability of avian coronavirus according to the mortality of SPF embryonated eggs and a Real-Time PCR for viral load estimation. This was a model to evaluate the sanitizing effects against coronaviruses since they share a very similar structure and chemistry with SAR-CoV-2. The influence of the type of textile treatment evidenced the potential effect of the sanitizing UV light, which achieved 100% of embryo viability. The response of the ZnONP + UV nebulization showed a notorious influence of photoactivation according to the exposure time, and the 60-s treatment achieved a decrease of 88.9% in viral viability, compared to 77.8% and 55.6% corresponding to the 120 and 180-s treatments, respectively. Regarding the decrease in viral load between the types of treatments, UV 180 s reduced 98.42% and UV 60 s + ZnONP reduced 99.46%, respectively. The results show the combinatorial effect of UV light and zinc nanoparticles in decreasing the viral viability of avian coronavirus, as a model of other important coronaviruses in public health such as SARS-CoV-2.

## Introduction

COVID-19 caused by the SARS-CoV2 virus emerged in December 2019 in Wuhan city, in Hubei province, China, in people exposed to wildlife animals traded in a public market^1^.

The family Coronaviridae comprises several genera of viruses causing respiratory and enteric disease in various avian and mammalian species, including man^1^. The study of these microorganisms, which affect both domestic and wild birds, is also important at the veterinary level^2^. Many of the studies currently carried out have been based on the study of old coronavirus variants, as in the case of the avian infectious bronchitis virus (gammacoronavirus)^3,4^.

Efforts to mitigate the impact of SARS-CoV-2 have been made in various areas of science such as nanotechnology^5^. Nanoparticles of various materials such as gold^6^, silver^7^, and titanium dioxide^8^ were used against various viruses showing the antiviral action of these materials at this scale. Likewise, studies show that zinc is a material that affects viral replication cycles^9^, while recent research shows how zinc oxide nanoparticles have an antiviral effect against the H1N1 influenza virus, having a virus inhibition rate of 52.2% at the concentration of 75 ug/mL^10^. Thus, it has also been shown that ZnO nanoparticles have an antiviral effect on the hepatitis A virus with percentages between 44.75 and 58.3% inhibition under maximum concentrations of non-toxicity^11^.

Nanoparticles are obtained by various mechanisms, and green synthesis is currently the most interesting methodology because it is eco-friendly, energy-efficient, and cost-effective compared to traditional chemical syntheses^5^. In green synthesis, the use of plant extracts allows obtaining nanoparticles due to the action of active plant compounds that act as reducing agents^12,13^. Thus, it was reported that the use of banana peel extract allowed synthesizing ZnO nanoparticles with a size of about 20nm^14^, while, using *Cinnamomum camphora* (L.) leaf extract, nanoparticles with a size between 13.92 and 21.13 nanometers were obtained, being confirmed by the use of transmission electron microscopy^15^. Similarly, using extracts of *Ficus carica* (*F. carica*)^16^ and *Cassia auriculata*^17^, zinc oxide nanoparticles between 30 and 40 nm were obtained, showing the reducing action of plant extracts in the synthesis of ZnO nanoparticles.

On the other hand, disinfection methods such as ultraviolet (UV) light have been developed. This is now considered a powerful disinfectant and is classified into UVA (320–400 nm), UVB (280–320 nm), and UVC (200–280 nm), being the UVC wavelength the most effective ultraviolet light used in clinical disinfection^18,19^. This has allowed knowing about several mechanisms of virus inactivation by UVC such as the damage generated in the genome of the influenza virus or the destruction of the capsid of the murine norovirus, among others. This diversity of induced effects shows the importance of performing new tests against coronaviruses, such as SARS-CoV-2^20–23^, to determine their antiviral action.

Given the importance of reducing the global impact caused by the COVID-19 pandemic, this study evaluates the antiviral effect of the treatment of textiles exposed to the avian infectious bronchitis virus, also known as avian coronavirus, using UV-C radiation and Zinc (ZnO) nanoparticles, with potential applications in the production of sanitizing systems.

## Methodology

### Green route-mediated phytosynthesis of ZnO nanoparticles

To obtain the ZnO nanoparticulate material (ZnONP), the green synthesis (phytosynthesis) method was considered, i.e., using as organic reductant the aqueous extract of *Coriandrum sativum*, starting from the precursor zinc acetate dihydrate (ZnC_4_H_6_O_4_) (Merck Millipore, Burlington, MA, USA, CAS no. 5970-45-6). For the extract, the leaves of *C. sativum* were washed with distilled water to remove any undesirable impurities, and then, dried in an oven (35 °C) for 10 h. This stage was concluded with the grinding and sieving. In the elaboration of the extract, 5 g of sieved powder of *C. sativum* were added to 83.33 mL ultrapure water, and the mixture was kept at 70 °C for 4 h in soxhlet extractor equipment and with magnetic stirring.

For the synthesis of ZnONP, 30 mL were prepared at a concentration of 0.21 M, and the mixture was brought to 70 °C in a hotplate, with magnetic stirring for 1 h. Then, 20 mL of the above-mentioned extract were added drop by drop and continuously stirred for 90 min; the temperature conditions were kept during the whole process. Finally, the obtained solution was calcined in a muffle (500 °C) for 2 h. A white powder was obtained, which was ground and washed three times with ultrapure water. For the application in the sanitizing locker, 400 mL of ZnONP were prepared at a concentration of 99.076 ppm. This research consolidates results where ZnONP obtained by green synthesis, activated by UV light and applied to textiles against avian coronavirus are applied for the first time, where the concentration value is due to the background of other works applied to various viruses^24–26^. Likewise, at the nanomaterial level it also depends on factors such as the morphology and size of the nanoparticles, in our case they are spherical and quite small, so 99,076 ppm would be within the average for their application in this field.

The electronic structure and optical properties of the nanoparticles were analyzed using UV vis spectrophotometry (Shimadzu, UV 1900, Tokyo, Japan), previously calibrated in the range from 370 to 800 nm. The particle size and shape of the nanoparticles in suspension was done using TEM (JEOL, JEM2011), at an acceleration voltage of 120 kV. Samples were prepared by placing 5 μl of the reaction mixtures on carbon-coated copper grids, followed by drying in a desiccator with silica for 16 h. EDX measurements were carried out with the TEM equipped with an OXFORD EDS 6498.

### Sanitizing locker operation

**Figure 1:**
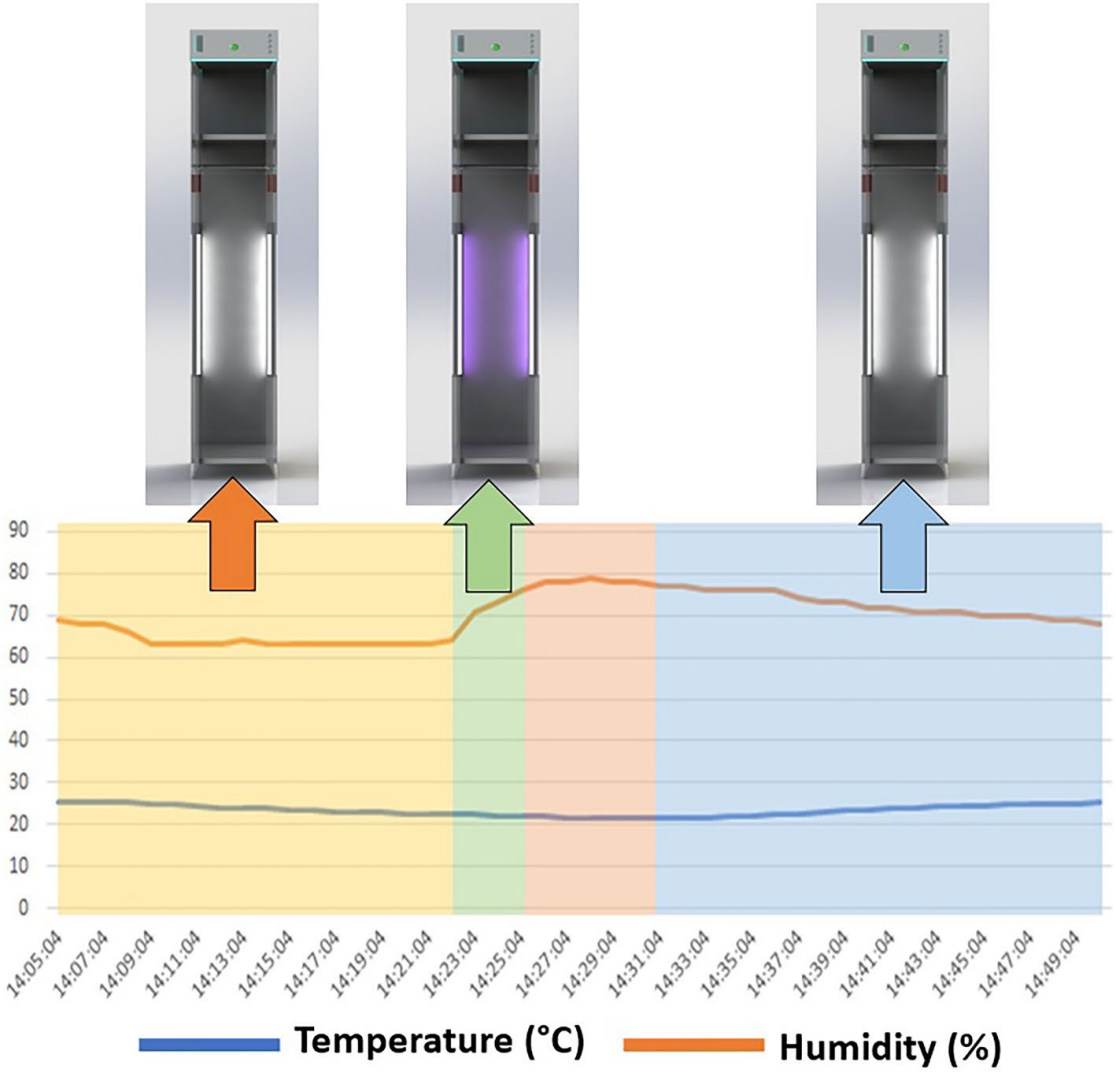
Temporal diagram of the sanitizing locker operation process.

The sanitizing locker was designed and built, which consisted of an ultrasonic nebulizer system located at the top, two electrical resistors at the base, and a tank with a volume of 500 mL located at the top, where the ZnONP colloid was stored. The research was carried out based on three types of treatments (UV only, ZnONP nebulization + UV, and Water nebulization + UV), and each treatment was linked to three different times (60, 120, and 180 s). Several factors were considered for the process to ensure that—once the disinfection process was completed—the ZnONP would not remain suspended in aerosols. Table 1 and Fig. 1 show the temporal processes of the treatment, which consists of the stabilization phase (pre-nebulization), where the initial measurement of the system humidity was considered; and the nebulizer operation phase (in this phase the times mentioned above were modified); the stabilization phase (post-nebulization); and, finally, the dehumidification phase, where the fan and electrical resistor were put into operation to reach the initial humidity conditions of the booth.

**Table 1:** Sanitizing locker operation process.

Initial humidity	63%
Stabilization time (Pre-nebulization)	15 min
Nebulizer and UV operating time	1,2,3 min
Stabilization time (Post-nebulization)	3 min
Dehumidification time (fan + resistor)	20 min
Total processing time	39–41 min
Maximum humidity reached	78%

### Estimation of viral viability

#### Experimental animals and groups

For the study of viral viability, SPF (Specific Pathogen Free) chicken embryonated eggs of 9 to 10 days of age were used, which were sent to the avian pathology laboratory of the Faculty of Veterinary Medicine of the Universidad Nacional Mayor de San Marcos, and incubated for 24 h to be observed in case of impact of transport stress. All the experimental procedures were approved by institutional ethics committee of the Universidad Privada del Norte with document 009-2022-DIIRS-UPN and were carried out in accordance with relevant guidelines and regulations. Besides all methods were accordance with ARRIVE guidelines.

Three disinfection methods for textile pieces (70% cotton and 30% polyester) exposed to avian infectious bronchitis virus were evaluated. The design comprised 05 groups. Groups 1, 2, and 3 were evaluated at three times according to the time of exposure to the disinfectant: 60, 120, and 180 s (strata). The distribution of groups is detailed below:

- **Group 1:** Textiles contaminated with avian coronavirus and subsequently treated with UV radiation. This treatment was evaluated at three UV exposure times: 60, 120, and 180 s (positive control).
- **Group 2:** Textiles contaminated with avian coronavirus and subsequently treated with UV radiation and ZnONP colloid nebulization. This treatment was evaluated at three UV and ZnONP exposure times: 60, 120, and 180 s.
- **Group 3:** Textiles contaminated with avian coronavirus and subsequently treated only with sterile distilled water. This treatment was evaluated at three different times: 60, 120, and 180 s (effect of the fabric on the coronavirus) (negative control).
- **Group 4:** Textiles contaminated with avian coronavirus, without treatment (recovery test). In order to evaluate the effect of the textile on the virus (called "SE"), group 4 infected without treatment was considered, corresponding to specimens that were inoculated and placed in the disinfection booth without the application of any process (equipment turned off), being recovered after 60 s.
- **Group 5:** Untreated, non-contaminated textiles (embryo toxicity test of the fabric).

#### Preparation of textile inoculum and viral inoculation

Infectious Bronchitis virus strain M41 was used, which was propagated, titrated, and brought to a concentration of 10^3^ EID_50_/0.1mL to be used for textile contamination. The protocol described in the AAAP Manual (AAAP, n.d.) was used for viral titration.

A cotton textile sent to the laboratory by the research group of the Universidad Privada del Norte (UPN) was used. For the test, using gloves, pieces of 20 mm x 20 mm were cut with sterile scissors.

Within each stratum, three pieces of the textile were evaluated to be placed in the sanitizing locker, located in three zones (upper, middle, lower) by hanging them with sterile nylon thread and placing them at a certain height with a space of 25 cm.

The fabric pieces were wrapped in craft paper and sterilized in an autoclave at 121 °C and 103 kPa for 15 min, and, then, dried for 24 h. Subsequently, each experimental group was placed in a container (Petri dish 90 mm x 17 mm) in a laminar flow cabinet.

The three textile pieces were inoculated in each Petri dish with a volume of 0.1 mL of avian infectious bronchitis virus suspension, at a concentration of 10^3^ EID_50_/0.1 mL for each stratum and experimental group.

The inoculated fabrics were incubated in Petri dishes for 1 h at 37 °C temperature. Subsequently, the plates were kept at refrigeration temperature (4–8 °C) until they were used in the experiment.

#### Exposure to the sanitization process and viral viability

To carry out the exposure to the sanitizing methods, a disinfection booth, the UV-C Sanitizing Locker (200 nm–280 nm), and ZnONP (99.076 ppm) were used following the manufacturer's technical instructions.

The textiles were placed on the three levels inside the booth, hanging at a distance of 25 cm from each other.

After the sanitizing exposure in the locker, each textile was placed in a 15 mL centrifuge tube using sterile tongs and was carefully identified. Subsequently, 10 mL of PBS were added to each container as collection solution and they were stirred by vortex for 10 s 5 times. From this solution, two 2 mL tubes were placed in cryotubes and preserved in ultra-freezing (− 80 °C) for the subsequent quantification of viral load by real-time RT-PCR.

To determine the presence of viable virus by observing lesions, SPF embryonated eggs were used. A volume of 0.2 mL of each collected sample was inoculated into 3 SPF chicken embryonated eggs (3 replicates) via the allantoic cavity. The embryos were incubated at 37 °C for 144 h (6 days). The embryos were evaluated twice a day using an ovoscope to determine daily mortality. At the end of the incubation period, embryos and allantoic fluid were collected for complementary tests. The presence of lesions in the embryos was observed.

The characteristic lesions expected in infection by infectious bronchitis virus correspond to dwarfism and coiling of the embryo. Mortality is occasional. Data collection was based on the frequency of detection of lesions by stratum and by group, determining the capacity of the virus to develop the infection.

### Viral quantification by real-time RT-PCR

The allantoic fluid was collected from the groups exposed to UV light and UV light + ZnONP (99.076 ppm), the group of fabrics contaminated with coronavirus without exposure to any sanitizer and the group of fabrics without contamination, and RT-PCR was performed according to a previously described methodology^27^.

The analyses were based on the cycle Threshold (Ct) values in the amplification curve of the samples, which is calculated by extrapolating the infective dose concentration or viral load based on the concentration of the original inoculum (whose concentration in EID_50_ is known).

The treatments used in the viral quantification assay were 1 (fabric contaminated with avian coronavirus and exposed to UV light at 60 s, 120 s, and 180 s), 2 (fabric contaminated with avian coronavirus and exposed to UV light+ ZnONP at 60, 120, and 180 s), 4 (fabric contaminated with avian coronavirus, without sanitizing exposure, and recovered after 60 s), in which each treatment was worked in duplicate, and treatment 5 (fabric with no viral contamination and no sanitizing exposure).

### Statistical analyses

An analysis of variance was performed between treatments 1 (UV at 60, 120, and 180 s), treatment 2 (UV+ ZnONP at 60, 120, and 180 s), and treatment 4 (group of fabrics contaminated with avian coronavirus without sanitizer) with a significance of 95% and a *p* < 0.05.

### Ethics approval

All the experimental procedures were approved by institutional ethics committee of the Universidad Privada del Norte with document 009-2022-DIIRS-UPN and were carried out in accordance with relevant guidelines and regulations. Besides all methods were accordance with ARRIVE guidelines.

## Results

### Phytosynthesis-mediated ZnO nanoparticles

**Figure 2:**
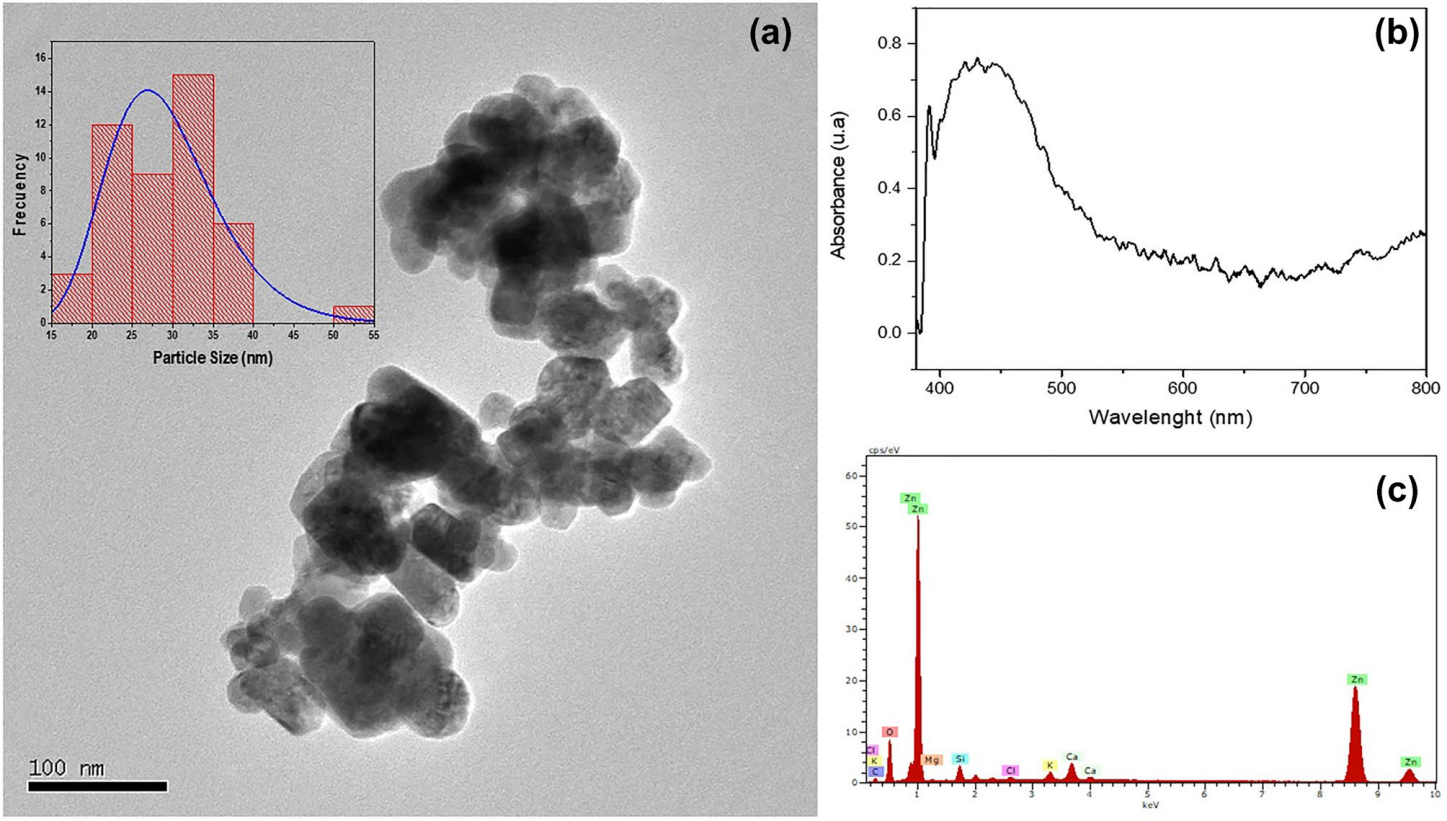
Characterization of ZnONPs. (**a**) Transmission electron microscopy shows the spherical morphology of ZnONPs with an average size of 30 nm. (**b**) UV vis spectrophotometry. (**c**) Elemental analysis by EDS.

The phytosynthesis method is shown as a potential alternative to the use of chemical-reducing compounds. Figure 2 shows the results of the colloidal characterization of the ZnONPs. Thus, it is possible to determine the spherical and rods morphology with an average size of 30 nm (Origin Software) (Fig. 2a). These results agree with other investigations in which similar morphologies have been obtained^28,29^. An important characteristic in nanoparticulate colloids is the verification of the surface plasmon resonance (SPR) peak. This is a response to the interaction of the electromagnetic wave and the density of the free electron plasma. It is worth mentioning that for this phenomenon to occur the particle has to be smaller than the wavelength of the incident light. Thus, Fig. 2b shows the UV vis spectrum with a peak at 391.2 nm, typical for this type of nanomaterial. Syntheses made with other methods show the presence of the absorbance peak in this range^30,31^. The elemental analysis by EDS (Fig. 2c) shows strongly defined peaks of zinc (Zn) within the composition of nanoparticles.

ZnONPs respond to UV radiation due to their semiconductor characteristic. Thus, this type of nanomaterial is shown to be a potential candidate to be applied in photocatalytic processes. The mechanism lies in the fact that the internal part of the ZnONP is usually quite active in the generation of holes and electrons, being a minimum those that migrate to the surface of the particle, allowing the absorption of oxygen molecules and OH^−^ ions which results in the high production of superoxide anion radicals (O^2−^) and hydroxyl radicals (OH), both immersed in a photocatalytic process. Their interaction regarding the avian coronavirus is possibly based on their capacity to generate free radicals damaging the viral protease and RNA polymerase.

### Viral viability

#### Viral viability analysis

The embryos were subjected to an incubation process of 144 h. One case of mortality within the first 24 h was observed in the Method 3 group. This embryo was not considered in the study. Likewise, mortality was found only in the Method 2 group (at the 6th day p.i.).

The following results were observed in each group analyzed:

**Method 1 Group**: (fabric contaminated with avian coronavirus and exposed to UV60, UV120, and UV180).

**Figure 3:**
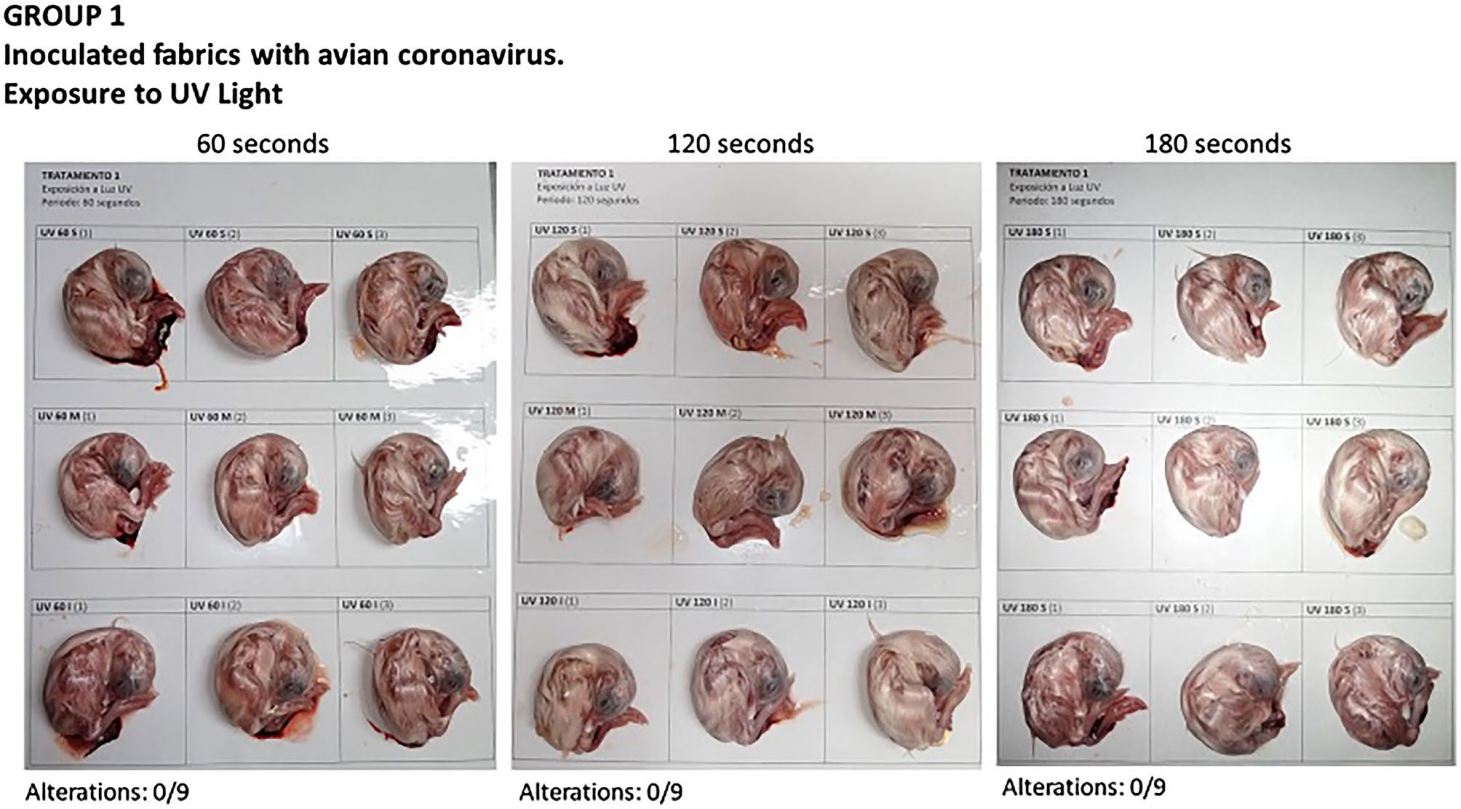
Embryos in group 1 in which the fabric was exposed to ultraviolet light and ZnONP at 60, 120, and 180 s, and where no alterations were observed in the embryos.

Embryo viability at 100% was obtained, as well as in each stratum of exposure time. No alterations related to a viral infectious process were observed. The embryos were apparently normal (Fig. 3).

**Method 2 Group:** (Fabric with ZnO NP dispersion and exposed to UV60-ZnONP, UV120-ZnONP, UV180-ZnONP).

**Figure 4:**
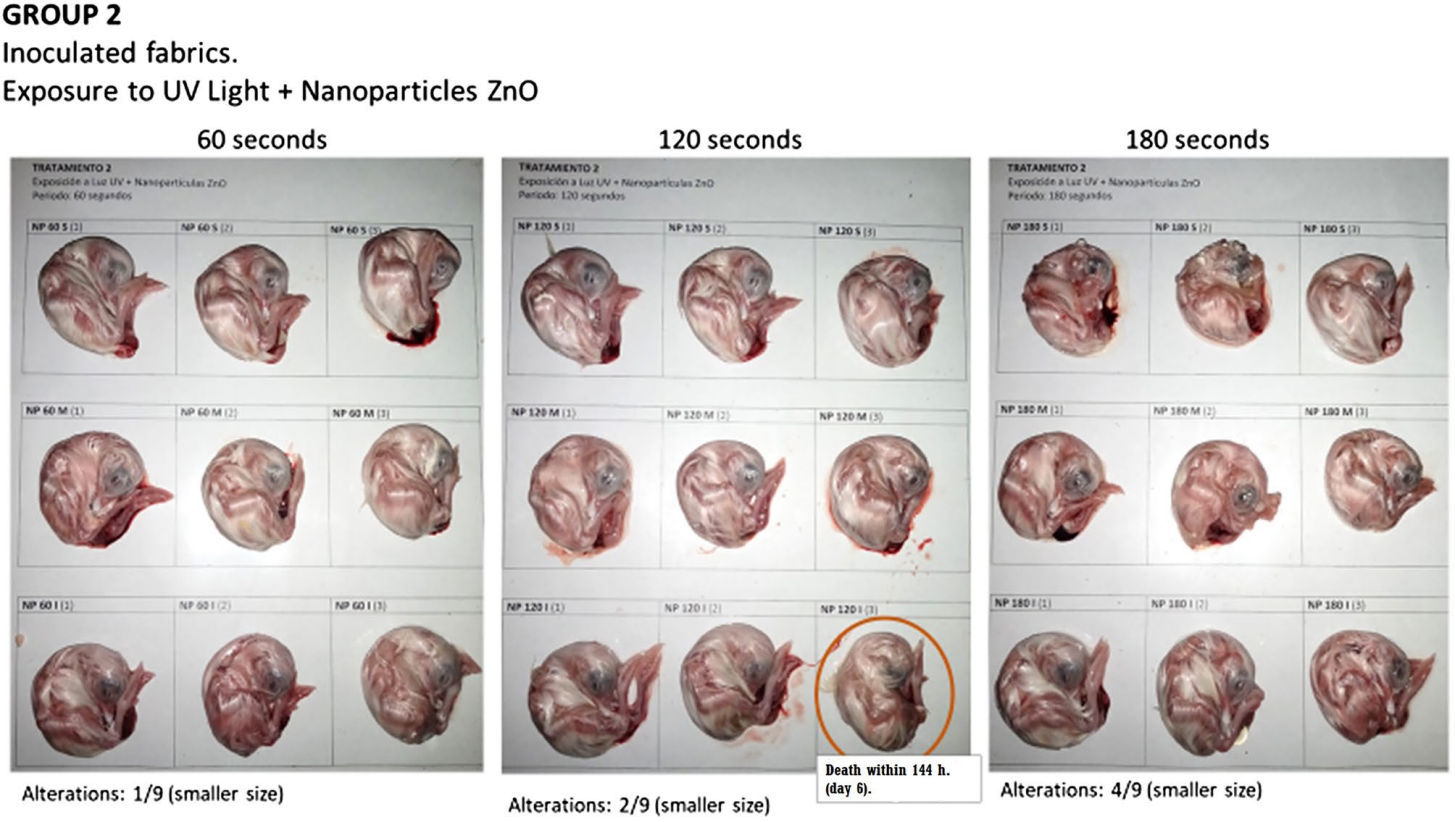
Embryo alterations related to avian coronavirus infection in group 2 in which the fabric was exposed to ultraviolet light and ZnONP.

Embryo viability was obtained at 100% in the strata corresponding to the exposure times of 60 and 180 s. In the case of the 120-s exposure time stratum, there was one death (88.9% embryo viability) (Fig. 4).

Alterations associated with a smaller embryo size were observed. In the 60-s stratum, 1/9 embryos of smaller size were observed (11.1%); in the 120-s stratum, 2/9 embryos (22.2%); and in the 180-s stratum, 4/9 embryos (44.4%).

In this group, there were also alterations in the chorioallantoic and amniotic membranes characterized by the presence of edema. This lesion was observed in all embryos in the 180-s exposure stratum (9/9 embryos), in the 120-s stratum, 3/9 embryos (33.3%) presented edema, and in the 60-s stratum, edema was observed in 2/9 embryos (22.2%).

**Method 3 Group:** (Fabric with distilled water dispersion and exposed to UV60, UV120, and UV180 seconds).

A 100% embryo survival rate was obtained. However, embryo alterations (smaller size, coiling, poor development) were observed in the different strata. In the 60-s stratum, alterations were observed in 5/8 embryos (62.5%); in the 120-s stratum, 7/9 embryos (77.8%); and, in the 180-s stratum, 7/9 embryos (77.8%).

**Method 4 Group**: (Fabric contaminated with avian coronavirus and recovered after 60 s):

In the group exposed to inoculated fabrics without exposure to sanitizing treatments, alterations associated with smaller size and coiling could be observed in 4/9 embryos (44.4%).

**Group 5**: White group (exposure to uninoculated fabrics):

To evaluate the effect of the textile on embryo viability, 9 embryos were inoculated with a solution recovered from uninoculated textiles. In this group, embryo alteration associated with smaller size was observed in 3/9 embryos (11.1%).

#### Controls

As a negative control, 5 embryos that did not undergo intervention were evaluated. In this group, no alterations were observed in the embryos (embryo viability test).

As a positive control, the original inoculum was inoculated in 4 embryos. In this group, characteristic alterations of dwarfism, body coiling, and poor development were observed in all embryos [alterations in 4/4 embryos (100%)] (viral viability test).

### Virus quantification

#### UV treatment

The analysis of the samples by real-time PCR, after the three exposure times, showed an increase in the CT values from 25.62 (obtained in the positive control of the study), to values between 30.12 and 31.24. These values indicated a reduction in viral load, which was greater as exposure time increased. UV treatment was able to reduce the genomic copy number of avian Coronavirus from 176.01 to 228.48 copies per 0.01 mL to 6.15 and 7.23 (at 60 s), to 4.53 and 4.0 (at 120 s), and to 3.06 and 3.33 (at 180 s). These values show a strong virucidal effect of UV treatment on avian Coronavirus, which increases with exposure time (Table 2).

**Table 2:** Quantification of the viral load in the allantoic fluid of embryos exposed to treated textiles.

Name	Ct	Genomic copies/0.01 mL (10 uL)	Result
Treatment 1-UV60—Repetition 1	30.33	6.15	Positive
Treatment 1-UV60—Repetition 2	30.12	7.23	Positive
Treatment 1-UV120—Repetition 1	30.73	4.53	Positive
Treatment 1-UV120—Repetition 2	30.89	4	Positive
Treatment 1-UV180—Repetition 1	31.24	3.06	Positive
Treatment 1-UV180—Repetition 2	31.13	3.33	Positive
Treatment 2-UV60-ZnONP—Repetition 1	32.58	1.09	Positive
Treatment 2-UV60-ZnONP—Repetition 2	32.56	1.11	Positive
Treatment 2-UV120-ZnONP—Repetition 1	31.54	2.43	Positive
Treatment 2-UV120-ZnONP—Repetition 2	31.32	2.88	Positive
Treatment 2-UV180-ZnONP—Repetition 1	31.37	2.77	Positive
Treatment 2-UV180-ZnONP—Repetition 2	31.85	1.92	Positive
Treatment 4 (Without exposure)—Repetition 1	25.62	228.48	Positive
Treatment 4 (Without exposure)—Repetition 2	25.96	176.01	Positive
Treatment 5 (Without contamination)			Negative
Positive IBV Control (Infectious Bronchitis Virus)	14.98	8.04 × 10^5^ copies	Positive

#### Treatment with UV + ZnO NP

The UV + ZnONP treatment showed an increase in CT values from 25.62 (obtained in the positive control of the study) to values between 31.32 and 32.58 (higher than those obtained with the UV treatment), indicating a greater virucidal effect of this treatment.

The UV + ZnONP treatment achieved a large reduction in the number of genomic copies of avian Coronavirus: from 176.01 to 228.48 copies per 0.01 mL to 1.09 and 1.11 (at 60 s), to 2.43 and 2.88 (at 120 s), and to 2.77 and 1.92 (at 180 s). These values show, independently of the exposure time, a strong virucidal effect of the UV + Zn NP treatment on avian Coronavirus (Tables 2, 3).

**Table 3:** Analysis of Variance between treatments.

Treatment	60 s	120 s	180 s
	Sig.	Sig.	Sig.
UV			
UV—ZnONP	1.00	1.00	1.00
Control	0.008	0.008	0.008
UV—ZnONP			
UV	1.000	1.000	1.000
Control	0.008	0.008	0.008
Control			
UV	0.008	0.008	0.008
UV—ZnONP	0.008	0.008	0.008
Between groups			
Sig. 0.004			

*The difference in means is significant at the 0.05 level.

Additionally, the Student's T-test between the treatments that obtained the best decrease in viral load by Real-Time PCR (UV 180 and UV60-ZnONP) was significant *P* < 0.05.

## Discussion

As observed, in the fabrics contaminated with avian coronavirus exposed to UV 60, 120, and 180-s radiation, 100% elimination of viral viability was achieved since all embryos exposed to the solution recovered from the contaminated fabric showed no pathology, in contrast to the treatment of the fabrics exposed to UV60-ZnONP, UV120-ZnONP, and UV180-ZnONP. The decrease in viral viability was inversely proportional to the exposure time UV60-ZnONP (88.9%), UV120-ZnONP (77.8%), and UV180-ZnONP (55.6%). It is likely that the exciton of the zinc oxide nanoparticles that generate free radicals and that are responsible for the rupture of the membrane in viruses has reached its peak before 120 s (Bindhu et al., 2020; Król et al., 2017), diminishing the effect of the exit due to the lack of electrons at as time goes by. In the groups of treatment with only distilled water, viral contamination without sanitizer, and only the use of the fabric without any contamination or exposure, it can be concluded that the fabric itself has a toxic effect on the embryos and the process of recovery of the avian coronavirus from the fabric influences the decrease in viral viability. Concerning the test to evaluate the decrease of viral viability, the best treatment was the exposure to ultraviolet light regardless of the exposure time, which was 60, 120, and 180 s. Regarding the UV + ZnONP treatment, the best assay was at 60 s, although it was less effective in its antiviral effect when compared to the UV light treatment. An interesting effect was the observation of edema in the embryos under treatment two, and this can be explained by the toxicity of zinc oxide at the assay concentrations (99.076 ppm).

As for virus quantification, a significant decrease in the viral load was observed for UV and UV + ZnONP treatments when compared to the control, which was treatment four with no sanitizing effect. However, when UV and UV + ZnONP treatments were compared at similar times, no significant difference was observed. In contrast, the best results among treatments for reducing the viral load were UV180 seconds (98.42%) and UV + ZnONP at 60 s (99.46%). A significant difference was observed, being the UV + ZnONP treatment at 60 s the best for reducing the viral load, although the sanitizing test was performed on a porous surface such as the fabric, which implies that the sanitizer may not reach the deepest parts of the fabric and present inconveniences in the process of viral recovery. The interaction between UV light and ZnO nanoparticles can generate reactive oxygen species (ROS), including free radicals, which are capable of damaging and oxidizing biomolecules such as proteins, lipids, and nucleic acids. This can have detrimental effects for viruses, such as viral inactivation and death. It is also important to mention that zinc oxide nanoparticles generating free radicals and therefore membrane rupture and destabilization of other molecules such as proteins, changing their charges and denaturing the molecules (Mohanan et al., 2017).

Nanoparticles are being used for different purposes against coronaviruses including diagnostic techniques and the removal of viral particles^32^. Although several studies have been developed to evaluate the sanitizing effect of various substances against SARS-CoV-2, avian coronavirus is a good model to evaluate the sanitizing effects against coronaviruses as they share with SARS-CoV-2 the chemistry and structure in which both viruses have an envelope, and are single-stranded, with positive-sense RNA^33–35^. Embryonated egg and RT-PCR models have been used before as a good system to evaluate viral viability and load of avian coronavirus^36^, in which avian coronavirus was evaluated when exposed to a detergent having a good virucidal effect, even though the sanitizing exposure was for 10 min^36^. In contrast, the maximum exposure time in this study was 3 min and the UV + ZnONP treatment at 60 s resulted in a 99.46% reduction of viral load and a decrease of viral viability by 88.9%. These findings indicate an effective antiviral effect of UV light + ZnONP, highlighting its sanitizing effect that could be used on surfaces to prevent outbreaks where the contamination of the viral agent is spread by contact with surfaces such as aprons or personal clothing, knowing that people may touch their mucous membranes between 10 to 23 times per hour after having been in contact with contaminated surface^37,38^.

Zinc oxide itself has already demonstrated an antiviral effect against SARS-CoV-2, Influenza A, and Herpes virus due to its capacity to generate free radicals damaging viral protease and RNA polymerase according to previous studies^39–42^. Thus, the antiviral effect reported in this study is verified. Among the limitations of this study, it can be mentioned that the assay at different concentrations of Zinc Oxide was not performed, nor was a toxicity assay performed, so it is recommended to perform this assay in future studies.

It is concluded that UV + ZnONP treatment at 60 s gave the best results in its antiviral effect regarding viral viability and viral load reduction.

## Conclusions

A correct sustainable synthesis mediated by the green route (phytosynthesis) was carried out, in which the extract of *C. sativum* acted as a reducing agent due to the presence of biomolecules acting in the formation of the ZnONP and obtaining spherical nanoparticles of 30 nm, thus consolidating the Zn^0^-phenolate complex by chelating effect. These nanoparticles were part of this research to consolidate UV light-mediated photoactivation processes in a sanitizing locker, which, in turn, were tested against UV and water + UV disinfection processes, besides evaluating the influence of exposure time. As it is known, UV light is a potential antimicrobial, which is proven in this research; however, it is worth noting that the ZnONP + UV treatment at 60 s showed the best results in its antiviral effect on viral viability and reduction of viral load.

## Data Availability

All data generated or analysed during this study are included in this published article.
